# Scurvy Outbreak Among South Sudanese Adolescents and Young Men — Kakuma Refugee Camp, Kenya, 2017–2018

**DOI:** 10.15585/mmwr.mm6803a4

**Published:** 2019-01-25

**Authors:** Mija Ververs, Jesse Wambugu Muriithi, Ann Burton, John Wagacha Burton, Allison Oman Lawi

**Affiliations:** ^1^Division of Global Health Protection, Center for Global Health, CDC; ^2^United Nations High Commissioner for Refugees, Kakuma, Kenya, Nairobi, Kenya, and Geneva, Switzerland; ^3^United Nations World Food Programme, Nairobi, Kenya.

Scurvy is a relatively rare micronutrient deficiency disease that can occur among refugees dependent on food aid (*1*). Inadequate access to fresh fruits and vegetables in refugee camps can result in scurvy (*2*,*3*). Kakuma Refugee Camp in Kenya’s Turkana District is home to 148,000 refugees, mostly from Somalia and South Sudan, who receive food assistance. In August 2017, a number of South Sudanese adolescent and young adult male refugees were evaluated at a health clinic in the camp for calf pain, chest pain, and gingival swelling. Because the symptoms were nonspecific, no diagnosis was made, and some patients received antibiotics and analgesics. All were managed as outpatients, but symptoms did not improve. During subsequent months, more young men with similar symptoms were reported. On January 20, 2018, the United Nations High Commissioner for Refugees (UNHCR) was informed and conducted clinical examinations. Signs and symptoms included lower limb pain and swelling (in some cases involving joints), lethargy, fatigue, gingival swelling and pain, hyperkeratotic skin changes, and chest pain. Based on these clinical findings, micronutrient deficiency, particularly vitamin C deficiency (scurvy), was considered a possible diagnosis, and an investigation of a possible outbreak was conducted. The suspected scurvy cases all occurred in young men from South Sudan who were living and cooking together in one geographic section of the camp. All patients who received treatment with vitamin C noted improvement of symptoms within <1 week. Patients were provided with food and cash assistance, the latter to allow dietary diversification (i.e., fresh fruits and vegetables). However, both forms of assistance were inadequate to allow access to sufficient amount of calories and the dietary diversification needed for intake of micronutrients, such as vitamin C. It is important to consider these needs when determining the amount of food or cash assistance provided to adolescents and young adult male refugees.

On January 26, 2018, serum specimens were collected from three of the patients with suspected scurvy, and test results indicated vitamin C levels of 2.89 mg/L, 3.06 mg/L, and 2.71 mg/L (normal = 2–14 mg/L); deficiency is defined as a vitamin C level <2mg/L (*1*). Levels of vitamins B1, B2, B6, and B12 in all three patients were normal. Although the serum vitamin C levels were within the low-normal range, these, in combination with the clinical signs and symptoms, suggested scurvy. Therefore, in February 2018, UNHCR requested assistance from CDC to investigate the suspected scurvy outbreak in the Kakuma camp.

## Investigation and Findings

Two health specialists from CDC and UNHCR conducted an outbreak investigation during March 11–17, 2018. A suspected scurvy case was defined as the occurrence of lower limb, knee joint, or ankle swelling, and at least two of the following signs or symptoms: calf pain, shin pain, knee joint pain, or gingivitis in a person of any age (*2*,*4*). Because the South Sudanese frequently have very dark skin, the typical dermatologic symptom of petechial hemorrhage was not included in the case definition.

Forty-five patients with suspected scurvy were identified and interviewed using a questionnaire developed by investigators to obtain information on symptoms and diet, with a recall period of 6 months. For a subset of 14 patients, the age structure of the household was analyzed. Additional interviews were conducted with staff members from UNHCR; the World Food Programme (WFP); the nongovernmental organization responsible for health care in the camp; community health volunteers; community leaders; and food shop owners who interacted with the patients. Dietary intake was estimated using WFP’s information on provided food rations and NutVal 4.1, a free software program for calculating the nutritional content of food rations (www.nutval.net/).

At the time of this investigation, all refugees in Kakuma received food assistance, consisting of cereal, pulses, fortified corn-soy blend (CSB+),* and vitamin A–fortified oil. By WFP standards, a food ration should provide 2,100 kcal per person per day (pppd), but after 2015, a part of the cereal component of the ration was replaced by electronic cash (e-cash)^†^ to provide dietary diversification and choice. In 2017 and 2018, one-person households received a 500 Kenyan Shillings (KSh)/pppd and food ration of 900–1,400 kcal/pppd. Households of ≥2 persons received 300 KSh/pppd and a food ration of 900–1,700 kcal/pppd.^§^ The variations in the food assistance from 2015 onwards resulted from shortages of commodities and funding shortfalls.

**FIGURE 1:**
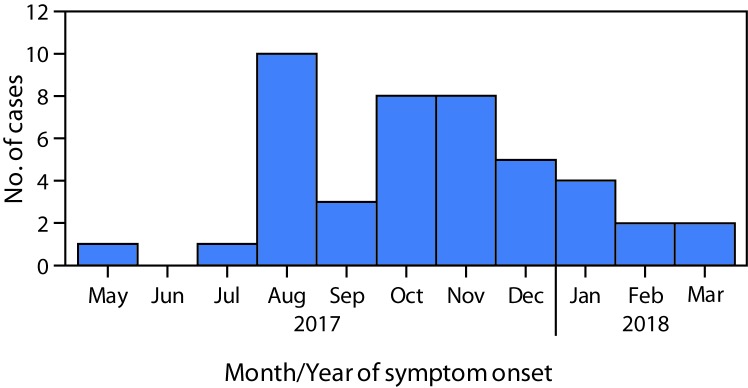
Suspected scurvy cases among South Sudanese refugees (N = 45),* by month and year of symptom onset — Kakuma Refugee Camp, Kenya, May 2017–March 2018 * Date of symptom onset was missing for one patient.

**FIGURE 2:**
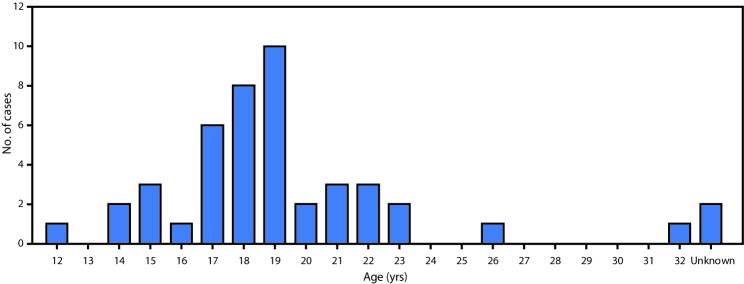
Age distribution of patients with suspected scurvy (N = 45) — Kakuma Refugee Camp, Kenya, 2017–2018

**FIGURE 3:**
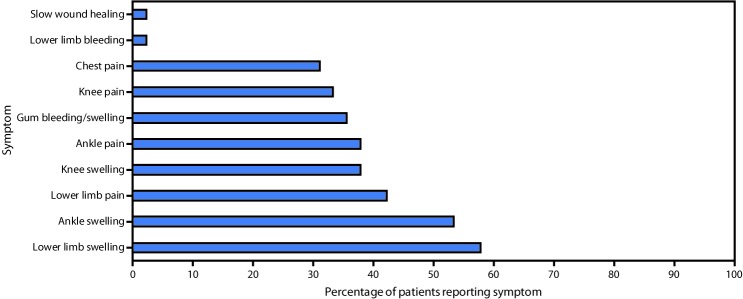
Percentage of South Sudanese refugees with suspected scurvy (N = 45), by selected reported symptoms* — Kakuma Refugee Camp, Kenya, 2017–2018 * Patients could report multiple symptoms.

Among the 45 patients with suspected scurvy, date of symptom onset was known for 44; among these, 29 (66%) reported onset during August–November 2017 (Figure 1). All 45 patients with suspected scurvy were adolescent and young adult male refugees from South Sudan who had arrived in Kakuma during 2012–2017; 33 (73%) had arrived in 2014 or later. The median age was 19 years (range = 12–32) (Figure 2). Approximately 58% of patients reported swelling of the lower limb, 53% ankle swelling, and 42% lower limb pain (Figure 3). Interviews with health personnel and patients found that approximately seven to 10 patients had been unable to walk. Forty of the 45 patients with suspected scurvy were treated with vitamin C.

The median household size of patients with suspected scurvy was five persons (range = one to 15). Among the subset of 14 households for which age was collected, nine (64.3%) included only adolescents and young men aged 13–22 years; only five households included a female, only one of whom was an adult.

All patients with suspected scurvy reported that they ate one meal per day. None had income from work or received any remittances, and all reported that rather than using the e-cash to diversify their diets, they used the full e-cash amount to purchase staple foods (e.g., cereals and pulses) and sometimes salt. Forty-three (96%) patients reported that they had not purchased vegetables, fruits, or potatoes since their arrival in Kakuma and used the e-cash to supplement their diet with cereals and pulses, which provided an additional 870–1,450 kcal/pppd.^¶^

All patients who received treatment with vitamin C** noted improvement of symptoms within <1 week, particularly reduction in swelling of knee and ankle joints and shin pain. All patients who previously had been unable to walk were able to do so after treatment.

In response to this outbreak, in April 2018, WFP tested the amount of vitamin C in CSB+, after simulating the CSB+ preparation in a laboratory setting. The raw product contains 90 mg vitamin C per 100 g, and each refugee received 40 g CSB+ per day (equivalent to 36 mg vitamin C per day). The cooking simulation demonstrated that vitamin C retention after preparation was <16%; thus, intake through consumption would be <6 mg vitamin C per day, which is insufficient to prevent deficiency (Food Safety and Quality Unit, World Food Programme, unpublished data, 2018).

## Discussion

In 2017, an outbreak of scurvy was identified in Kakuma Refugee Camp; the diagnosis was based on clinical manifestations, dietary history, and response to treatment. Although chest pain is not often described as a symptom of scurvy in this age group, it was frequently reported in this outbreak. This pain is believed to have resulted from the effect of scurvy on the collagen-containing cartilage in the distal rib ends (costochondral junctions). The actual date of onset of the outbreak remains unknown, but there was an increasing number of cases during August–November 2017. The outbreak was ongoing in early March 2018, although the number of cases had declined.

Scurvy is not new to refugee settings in which a limited amount of fresh foods is available or affordable and has previously been documented in Kakuma Refugee Camp, with outbreaks reported during 1995–1997 (*5*) and in 2003 (*6*). Vitamin C deficiency has also been described among refugees and imprisoned male populations in similar geographic areas (*2*–*4*,*7*).

The energy requirements for males aged 14–18 years and 18–30 years are 3,000–3,400 kcal per day and 2,550–3,900 kcal per day, respectively (*8*), based on moderate physical activity (males aged 14–18 years) and active to moderately active physical activity (men aged 18–30 years). The food ration provided in the camp supplied 900–1,700 kcal/pppd; with all e-cash used to purchase sorghum and split peas, an additional 870–1,450 kcal/pppd was potentially available, for a maximum theoretical intake of 1,800–2,900 kcal/pppd, depending upon household size. Thus, the food ration met only half of the required caloric needs. Because the e-cash intended for dietary diversification was not used to purchase fresh foods, such as vitamin C–rich vegetables and fruits, but rather to complement the food rations with more calorically dense and cheaper staple foods to secure the missing calories, vitamin C deficiency resulted. The diet of patients with suspected scurvy contained, on average, <10 mg vitamin C per day, which is insufficient to prevent scurvy (*1*). Despite previous assumptions, the fortified commodity, CSB+, was not a sufficient source of vitamin C as losses during preparation were much higher than initially estimated (*9*,*10*). The geographic clustering of suspected cases likely resulted from the relatively higher number of young men living and cooking together in one area of the camp and sharing their limited food rations and e-cash.

The findings in this report are subject to at least two limitations. First, symptoms were self-reported. Second, the investigation took place in the aftermath of the outbreak. The focus was on identifying the cause of the outbreak and possible solutions.

Provision of food assistance in refugee settings is often based on average household composition, factoring in age, sex, and caloric needs. In this investigation, the adolescent and young males had very high nutritional needs compared with persons in an average household. These differences in household demographics demonstrate that simply providing an average amount of calories calculated on assumed household demographics is inadequate to meet nutritional requirements. In addition to food rations, refugees were provided with e-cash to purchase their own food to add diversity and choice to their diet. However, this investigation indicated that for adolescent and young adult male refugees, both forms of assistance were inadequate to allow access to sufficient amount of calories and the dietary diversification needed for intake of micronutrients, such as vitamin C. It is important to consider these needs when determining the amount of food or cash assistance provided to adolescents and young adult male refugees.

**Summary**

**What is already known about this topic?**

Inadequate access to fresh fruits and vegetables in refugee camps can result in scurvy.

**What is added by this report?**

An outbreak of scurvy occurred among adolescent and young adult male South Sudanese refugees who had been provided electronic cash to supplement their diets. However, rather than purchasing fresh foods rich in vitamin C but lower in calories, they selected more calorie-dense cereal and pulses to meet their caloric needs. Symptoms resolved after vitamin C treatment.

**What are the implications for public health practice?**

The type of food purchased with electronic cash might not meet caloric and micronutrient needs. Providing the standard provision of 2,100 kcal/person/day is insufficient for refugees with higher caloric needs, and it is important to consider these needs when determining the amount of food or cash assistance provided to adolescents and young adult male refugees.

## Footnotes

* Mix of corn and soy flour fortified with micronutrients.

† Bamba Chakula or “get your food” recipients were instructed to buy their own (preferably nutritionally adequate) food.

§ 100 KSh = approximately 1 USD.

¶ If all e-cash is transformed into food (two thirds sorghum and one third split peas); 300 Ksh provides 190 g sorghum grain/pppd and 67 g of split peas/pppd, amounting to 870 kcal/pppd; 500 Ksh provides 317 g sorghum grain/pppd and 110 g of split peas/pppd amounting to 1,450 kcal/pppd.

** Oral treatment with vitamin C had been provided by local health clinic in dosages varying from 200 mg to 300 mg daily for 1–3 weeks. However, the investigation resulted in the change in dosage to 600 mg per day for 3 days, followed by 200 mg per day for 11 days.
